# The Model for Early COvid-19 Recognition (MECOR) Score: A Proof-of-Concept for a Simple and Low-Cost Tool to Recognize a Possible Viral Etiology in Community-Acquired Pneumonia Patients during COVID-19 Outbreak

**DOI:** 10.3390/diagnostics10090619

**Published:** 2020-08-21

**Authors:** Gianluca Sambataro, Mauro Giuffrè, Domenico Sambataro, Andrea Palermo, Giovanna Vignigni, Roberto Cesareo, Nunzio Crimi, Sebastiano Emanuele Torrisi, Carlo Vancheri, Lorenzo Malatino, Michele Colaci, Nicoletta Del Papa, Francesca Pignataro, Erik Roman-Pognuz, Massimiliano Fabbiani, Francesca Montagnani, Chiara Cassol, Lorenzo Cavagna, Valentina Zuccaro, Verena Zerbato, Cristina Maurel, Roberto Luzzati, Stefano Di Bella

**Affiliations:** 1 Department of Clinical and Experimental Medicine, Respiratory Medicine Unit, University Hospital “Policlinico-Vittorio Emanuele”, University of Catania, 95123 Catania, Italy; giovannavignigni@gmail.com (G.V.); crimi@unict.it (N.C.); torrisiseby@hotmail.it (S.E.T.); vancheri@unict.it (C.V.); 2 Department of Medical, Surgical and Health Sciences, University of Trieste, 34151 Trieste, Italy; gff.mauro@gmail.com (M.G.); verena.zerbato@gmail.com (V.Z.); cristina.maurel@hotmail.it (C.M.); roberto.luzzati@asugi.sanita.fvg.it (R.L.); stefano932@gmail.com (S.D.B.); 3 Italian Liver Foundation, Basovizza, 34149 Trieste, Italy; 4 Artroreuma S.R.L., Outpatient of Rheumatology Associated with the National Health System corso S. Vito 53, Mascalucia, 95030 Catania, Italy; d.sambataro@hotmail.it; 5 Department of Clinical and Experimental Medicine, Internal Medicine Unit, Cannizzaro Hospital, University of Catania, via Messina 829, 95100 Catania, Italy; malatino@unict.it (L.M.); michele.colaci@unict.it (M.C.); 6 Unit of Endocrinology and Diabetes, Campus Bio-Medico University, 00128 Rome, Italy; a.palermo@unicampus.it; 7 Unit of Metabolic Diseases, “S.M. Goretti” Hospital, 04100 Latina, Italy; robertocesareo@libero.it; 8 Dept Rheumatology, ASST Pini-CTO, Piazza Cardinal Ferrari 1, 20122 Milan, Italy; nicoletta.delpapa@asst-pini-cto.it (N.D.P.); francy.pignataro@hotmail.it (F.P.); 9 Department of Perioperative Medicine, Intensive Care and Emergency, University Hospital, 34151 Trieste, Italy; romanpognuz.erik@gmail.com; 10 Infectious and Tropical Disease Unit, Azienda Ospedaliero-Universitaria Senese, 53100 Siena, Italy; massimiliano.fabbiani@gmail.com (M.F.); francesca.montagnani@unisi.it (F.M.); chiaracassol92@gmail.com (C.C.); 11 Department of Medical Biotechnologies, University of Siena, 53100 Siena, Italy; 12 Rheumatology Division, University and IRCCS Policlinico San Matteo Foundation, Lombardia, 27100 Pavia, Italy; lorenzo.cavagna@unipv.it; 13 Infectious Diseases Clinic, University and IRCCS Policlinico S. Matteo Foundation, 27100 Pavia, Italy; v.zuccaro@smatteo.pv.it

**Keywords:** COVID-19, SARS-CoV-2, coronavirus, interstitial lung disease, diagnosis, triage, pneumonia, blood cell count, platelets, neutrophils

## Abstract

This study aims to assess the peripheral blood cell count “signature” of Severe Acute Respiratory Syndrome-Coronavirus 2 (SARS-CoV-2) to discriminate promptly between COronaVIrus Disease 19 (COVID-19) and community-acquired pneumonia (CAP). We designed a retrospective case-control study, enrolling 525 patients (283 COVID-19 and 242 with CAP). All patients had a fever and at least one of the following signs: cough, chest pain, or dyspnea. We excluded patients treated with immunosuppressants, steroids, or affected by diseases known to modify blood cell count. COVID-19 patients showed a significant reduction in white blood cells (neutrophils, lymphocytes, monocytes, eosinophils) and platelets. We studied these parameters univariately, combined the significant ones in a multivariate model (AUROC 0.86, Nagelkerke PSEUDO-R2 0.5, Hosmer–Lemeshow *p*-value 0.9) and examined its discriminative performance in an internally-randomized validation cohort (AUROC 0.84). The cut-off selected according to Youden’s Index (−0.13) showed a sensitivity of 84% and a specificity of 72% in the training cohort, and a sensitivity of 88% and a specificity of 73% in the validation cohort. In addition, we determined the probability of having COVID-19 pneumonia for each Model for possible Early COvid-19 Recognition (MECOR) Score value. In conclusion, our model could provide a simple, rapid, and cheap tool for prompt COVID-19 diagnostic triage in patients with CAP. The actual effectiveness should be evaluated in further, prospective studies also involving COVID-19 patients with negative nasopharyngeal swabs.

## 1. Introduction

The outbreak of the novel COronaVirus Disease 2019 (COVID-19) caused by the Severe Acute Respiratory Syndrome-CoronaVirus 2 (SARS-CoV-2) began in December 2019. Since then, the disease has caused a global pandemic, and as of 26 June 2020, the World Health Organization (WHO) reported about 10 million cases of COVID-19 worldwide with approximately 500,000 deaths. Given the current emergency, clinicians and researchers are looking for new tools that could allow the early identification of COVID-19 patients [1]. Currently, the diagnosis is made through rhino-pharyngeal swabs, even considering the high risk of false negatives [2]. Furthermore, other techniques, such as viral detection in sputum, tracheal-aspirate, and Broncho-Alveolar Lavage (BAL) [3] may be more sensitive, but they are not yet routinely used. Finally, serological assays for immunoglobulin [4] produce information regarding contact with the virus, rather than an actual, current infection, and they are not rapidly available. In addition, imaging techniques are not always able to confidently distinguish COVID-19 from other pneumonia etiologies.

In recent years, growing evidence has supported the role of the simple blood cell count in predicting prognosis in several conditions. These studies evaluated the role of simple, rapid, nonexpensive tools such as Neutrophil-to-Lymphocyte Ratio (NLR). This index showed good results in predicting mortality, disease activity, and severity in a broad spectrum of diseases such as Community-Acquired Pneumonia (CAP), atherosclerosis, cancer, and autoimmune disorders [5,6,7,8,9,10,11]. In particular, a high NLR was found to predict mortality [5] and admission to a respiratory intensive care unit [9] in patients with CAP.

Several studies reported alterations in peripheral blood cells in COVID-19 patients and their association with disease severity. High ratio of NLR and lymphopenia are associated with worse outcomes, whereas an increased level of eosinophils could positively affect the final prognosis [12,13,14,15]. That being said, the use of a simple white blood cell count to distinguish between COVID-19 and CAP that could show similar clinical presentations has not been explored yet.

The current study aims to evaluate the potential role of the complete blood cell count in predicting the presence of SARS-CoV-2 infection in patients presenting with pneumonia symptoms.

## 2. Materials and Methods

This is a case-control study involving patients with COVID-19 and CAP as the control group. The study was conducted retrospectively, including COVID-19 symptomatic patients with positive swabs admitted between February and April 2020 in Italy from participating centers. We decided to use CAP as the control group to compare between COVID-19 and a common disease mimicker with different management in terms of therapy and patients’ isolation. This study was conducted according to the declaration of Helsinki approved by the Ethics Committee (Unique RegionalEthical Committee Friuli Venezia-Giulia 16 April 2020 cod. CEUR 2020-OS-072).

The diagnosis of COVID-19 was made through suggestive symptoms, positive swabs, and consistent imaging in chest radiography and/or Computed Tomography.

Considering that the prevalence of CAP is estimated at 258 patients in every 100,000 individuals, considering a confidence interval of 95% and a confidence interval of 7, we calculated an expected sample size of 196 individuals of CAP. In addition, as of 30th April 2020, 18,000 patients with COVID-19 were hospitalized in Italy. With that taken into consideration, considering a confidence level of 95% and a confidence interval of 7, we calculated a sample size of 194 subjects with proven COVID-19 infection that required hospitalization.

### 2.1. Inclusion Criteria

We included consecutive adult patients (>18 years) with a fever ≥37.3 °C, presenting dyspnea, and/or chest pain or cough requiring hospitalization and undergoing a full white blood cell (WBC) count on admission, which included neutrophils (NEUT), lymphocytes (LYM), monocytes (MON), eosinophils (EOS), and platelets (PLT). Patients must have been diagnosed with CAP (as reported on hospital discharge charts before the SARS-CoV-2 outbreak, January 2017–December 2018) or with SARS-CoV-2 infection, which was confirmed by real-time reverse-transcription polymerase-chain-reaction (RT-PCR) assay from nasal and pharyngeal swabs.

### 2.2. Exclusion Criteria

Patients were excluded from the study for (1) missing data regarding complete blood count, chest imaging, clinical presentation, pharmacological anamnesis, and clinical history for previous and concurrent comorbidities: (2) current hospital-acquired pneumonia; (3) lung malignancies (both primary and metastatic neoplasms); (4) history of chemotherapy; (5) current use of steroids or treatment with any immunosuppressive drug; (6) known HIV infection; (7) liver cirrhosis; (8) organ transplantation; (9) mild pneumonia not requiring hospitalization.

### 2.3. Patient Data Collection

Data collection of the training and validation cohorts was conducted retrospectively, involving patients with symptoms suggestive of pneumonia and referred to five hospital centers, each of them located in distinct territories of North, Central, and South Italy. In particular, patients with CAP were evaluated between January 2017 and December 2018, before the SARS-CoV-2 outbreak. On the contrary, data regarding the SARS-CoV-2 infection were collected from patients admitted to the five participating centers between February and April 2020. The patients mentioned in the results have already been screened by the referring center for the application of inclusion/exclusion criteria.

### 2.4. Statistical Analysis

Variables were reported as the median and interquartile range (IQR, 1–3). We explored intercohort (training cohort vs. validation cohort) and intracohort (SARS-CoV-2 negatives vs. positives) differences using the Mann–Whitney *U* test or the Wilcoxon Sum-Rank test for continuous variables and the Pearson’s Chi-Square Test for discrete variables.

The variables that turned out to be statistically different between SARS-CoV-2 negative and SARS-CoV-2 positive patients in the training cohort were modeled in a univariate fashion using a binary logistic regression. In particular, the probability of testing positive for SARS-CoV-2 infection was calculated using SARS-CoV-2 diagnosis as the dependent variable (0 = non-SARS-CoV-2 pneumonia; 1 = SARS-CoV-2 pneumonia) and white blood cell parameters. Models were compared regarding their discrimination (i.e., the ability of the studied model to distinguish correctly between the two classes of outcomes) [16] and calibration (i.e., the measure of how close the predicted probabilities are to the observed rate of the selected outcome for any given value of the independent variable that constitutes the model) [16,17], through the area under the receiver-operating characteristic curve (AUROC), Akaike information criterion (AIC), the Bayesian information criterion (BIC), the Hosmer–Lemeshow goodness-of-fit test, and the Nagelkerke pseudo-R2. A complete assessment of model performance should take into consideration both discrimination and calibration. Therefore, we estimated a multivariate model including those variables that univariately showed an AUROC ≥ 0.65 and a Hosmer–Lemeshow *p*-value ≥ 0.05. The multivariate model was estimated through forward stepwise imputation (*p* < 0.10 to enter, *p* > 0.15 to be removed). Additionally, before being included in the model, variables underwent a nonlinear transformation using natural logarithm or arctangent functions, which were performed to obtain a linear relationship between the log odds and the independent variable and to obtain a final score that could range between −10 and +10. Between the seven possible multivariate models that could be obtained, we chose the one that showed the lowest AIC and where the single variable showed a variance inflation factor (VIF) ≥ 5, to exclude residual multicollinearity [18]. The linear predictor (LP) of the multivariate model was calculated as the sum of the intercept (β_0_), and each coefficient multiplied for the related variable (β_n_ × *x*_n_). For the sake of simplicity, the LP of the multivariate model was renamed as the Model for possible Early COvid-19 Recognition (MECOR) Score. To obtain the probability of being SARS-CoV-2 positive according to the MECOR Score, the LP was computed in the following function:

(1) $$f\left(LP\right)=1-\left(\frac{1}{1+\;e^{LP}}\right)$$

To validate our results, we randomly selected an internal derivative cohort of 250 patients maintaining the ratio of 1:16 (COVID-19/CAP) found in the training cohort.

Optimal MECOR Score cut-off values were chosen in the training cohort according to Youden’s Index [19], or to either maximize sensitivity or specificity. The same cut-offs were also applied in the validation cohort, after AUROC computation and inter-ROC curve difference analysis employing the Hanley–McNeil Test [20]. Cut-off characteristics were reported using sensitivity, specificity, positive predictive value (PPV), negative predictive value (NPV), accuracy, positive likelihood ratio (+LR), and negative likelihood ratio (−LR). For all analyses, two-sided statistical significance was defined as *p* < 0.05. Data were analyzed using SPSS (Statistical Package for Social Science) version 26.0 (IBM SPSS Statistics for MAC OS. Armonk, NY, USA: IBM Corp.).

## 3. Results

We collected data from 525 patients in the training cohort, of which 283 (53.9%) had COVID-19, and 242 (46.1%) were diagnosed with CAP (control group). The identified CAP pathogens are reported in Table 1. Our CAP cohort included 30% of interstitial pneumonia. Three-hundred and thirty-one (63%) patients were male with a median age of 64 (52–77) years. In the validation cohort, we collected data from 250 patients (135 COVID-19, 115 control group), who were predominantly male (*n* = 164, 65.6%) and had a median age of 63 (53–77) years. As auspicated, there was no statistically significant difference in blood leukocytes and SARS-CoV-2 infection prevalence between the two cohorts (Table 1).

In the training cohort, SARS-CoV-2 positive patients had lower median WBC (5930 vs. 11,465 cells/mm^3^, *p* < 0.00001), NEUT (4300 vs. 8925 cells/mm^3^, *p* < 0.00001), LYM (830 vs. 1185 cells/mm^3^, *p* = 0.005), MON (430 vs. 755 cells/mm^3^, *p* < 0.00001), EOS (0 vs. 20 cells/mm^3^, *p* < 0.00001), and PLT (186,000 vs. 251,000 cells/µL, *p* < 0.00001) when compared to patients with CAP. A similar trend was also observed in the validation cohort as shown in Table 2.

### 3.1. Model Analysis

**Figure 1:**
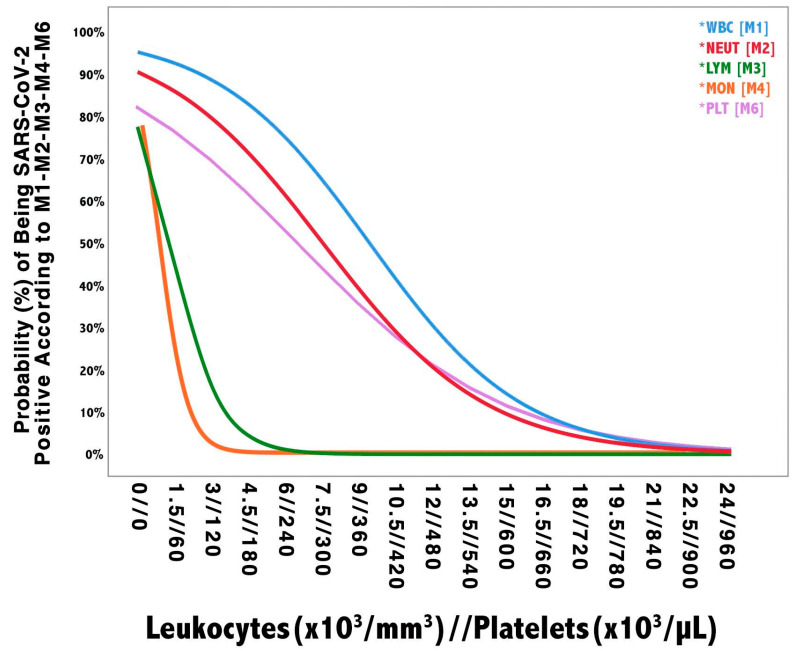
Univariate models probability plot. The vertical axis shows the probability of being SARS-CoV-2 positive according to each model. The horizontal axis shows the values of blood leukocytes (i.e., WBC, NEUT, LYM, and MON) and platelets, which are reported as leukocytes//platelets. Given the different amplitude of leukocytes and platelets blood concentration, their respective units of measure, as reported in the figure, should be multiplied by a factor of 10^3^ (e.g., 1.5 leukocytes × 10^3^/mm^3^ are equivalent to 1500 cells/mm^3^ and 60 platelets × 10^3^/µL are equivalent to 60,000 cells/µL). WBC: white blood cell; NEUT: neutrophils; LYM: lymphocytes; MON: monocytes; EOS: eosinophils; PLT: platelets.

As explained in the methods section, we initially built six univariate models (Model 1 through 6) based on variables that resulted significantly different between SARS-Cov-2 negative and positive patients in the training cohort. As reported in Table 3, M1, M2, M3, M4, and M6 showed an AUROC ≥ 0.65 and a Hosmer–Lemeshow *p*-value ≥ 0.05. The probability of being SARS-CoV-2 positive according to changes in the variable constituting M1, M2, M3, M4, and M6 is plotted in Figure 1. Then, we proceeded to the nonlinear transformation of the independent variables of M1, M2, M3, M4, and M6 as explained in the method section. Transformed variables were computed in a multivariate model (MM). The MM (Table 4) showed the following metrics: AUROC 0.86, Nagelkerke PSEUDO-R2 0.5, Hosmer–Lemeshow *p*-value 0.9, AIC 516, BIC 533). For the sake of simplicity, the LP of the MM was renamed as the “MECOR Score”, and its formula is reported below:

(2) $$\mathrm{MECOR}\;\mathrm{Score}\;=\;18.47\;-\;3.23\times \log_{e}\left(\mathrm{WBC}\left[\mathrm{cells}/{\mathrm{mm}}^{3}\right]\right)\;-\;0.76\;\times \log_{e}\left(\mathrm{LYM}\left[\mathrm{cells}/{\mathrm{mm}}^{3}\right]\right)\;+\;11.94\;\times \arctan\left(\frac{\mathrm{MON}\left[\mathrm{cells}/{\mathrm{mm}}^{3}]\;\times \;\mathrm{NEUT}[\mathrm{cells}/{\mathrm{mm}}^{3}\right]}{\mathrm{PLT}\left[\mathrm{cells}/\mu L\right]}\right)$$

By replacing patients’ variables, with the same unit of measure as reported in the formula, we performed the canonical cut-off analysis, which is reported in Table 5. In particular, (1) a MECOR Score ≤ −2.54 showed a 100% sensitivity, 10% specificity, 56% PPV, 100% NVP, 1.11 +LR, and 59% accuracy; (2) a MECOR Score ≥ −0.13 showed an 84% sensitivity, 72% specificity, 78% PPV, 79% NVP, 2.92 +LR, 0.23 −LR, and 80% accuracy; (3) a MECOR Score ≥ +4 showed a 2% sensitivity, 100% specificity, 100% PPV, 47% NVP, 0.98 −LR, and a 47% accuracy.

**Figure 2:**
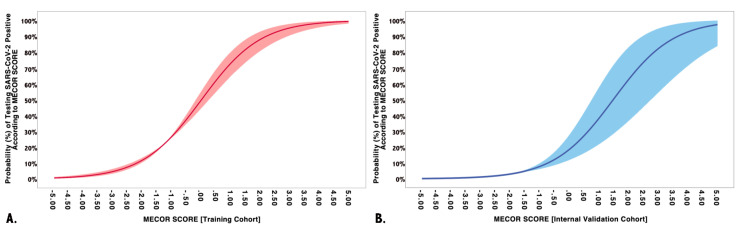
Multivariate model performance in training and validation cohorts. (**A**) The training cohort on the vertical axis represents the probability of being SARS-CoV-2 positive according to the Model for possible Early COvid-19 Recognition (MECOR) Score (horizontal axis). Logistic regression coefficients of probability are represented with robust 95% confidence intervals (light red area); (**B**) the validation cohort on the vertical axis represents the probability of being SARS-CoV-2 positive according to the MECOR Score (horizontal axis). Logistic regression coefficients of probability are represented with robust 95% confidence intervals (light blue area).

However, we proceeded further by determining the probability of testing positive to SARS-CoV-2 according to the MECOR Score, employing the formula reported in the method section, whose graphical representation is plotted in Figure 2. The slope of the probability curve sharply rose from a MECOR Score equal to −3 and reached a plateau at a MECOR Score of +4. This behavior can be interpreted as follows: the probability of being SARS-Cov-2 positive was approximately 0% for values < −3, then, it steadily increased and reached 100% for values > 4.

### 3.2. MECOR Score Application in the Validation Cohort

**Figure 3:**
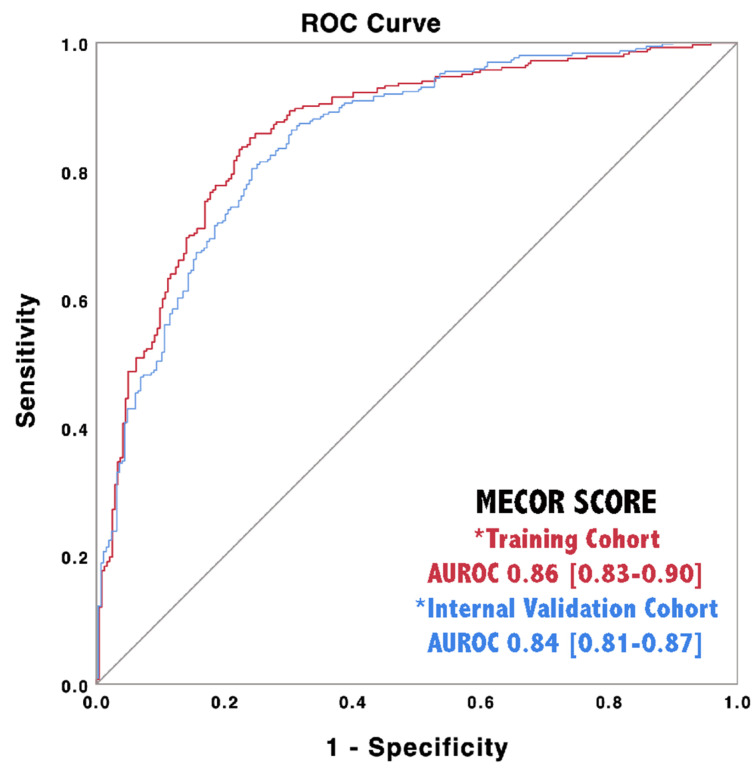
Discriminative ability of the MECOR Score in the training cohort (red line) and in the internal validation cohort (blue line).

We applied the MECOR Score to the validation cohort and evaluated its discriminative ability through AUROC analysis. As reported in Figure 3, the MECOR Score showed an AUROC of 0.84 (95% Confidence Interval, CI 0.81–0.87), comparable to the training cohort (nonsignificant Hanley–McNeal test). Afterward, we applied the cut-offs selected for the training cohort, and their characteristics are reported in Table 5. Additionally, we proceeded further by plotting the probability of being SARS-Cov-2 positive according to the MECOR Score in the validation cohort (Figure 2). If compared to the probability plot of the training cohort, it appears to be translated towards the right. In particular, the slope of the probability curve sharply rose from a MECOR Score equal to −1 and reached a plateau at a MECOR Score of 5. This behavior can be interpreted as follows: the probability of being SARS-Cov-2 positive was approximately 0% for values < −1, then it steadily increased and reached 100% for values > 5.

### 3.3. Online Calculator

An online calculator and a mobile app based on the MECOR Score were developed to allow clinicians to enter the values of the five variables required to obtain the probability of being SARS-CoV-2 positive based on admission laboratory tests (http://bit.ly/mecorscore).

## 4. Discussion

In line with previous studies [12,13,14,15], we found significant differences in peripheral blood cells count between CAP and COVID-19 pneumonia, with a reduction in all White Blood Cell (WBC) populations and platelets. In particular, lymphopenia and low eosinophil count could reliably predict worse outcomes if compared to other serological parameters [21]. However, the explanation of these associations, and the “peripheral withe blood signature” of SARS-CoV-2 is currently mostly speculative.

### 4.1. Behind the Pathophysiology of COVID-19 White Blood Cell Signature

The proposed mechanisms able to explain lymphopenia are related to several mechanisms, such as direct infection through the receptor ACE2, a potential direct attack on the lymphatic organs, lymphocytic apoptosis due to the exposition to high levels of Tumour Necrosis Factor-α and Interleukin-6 or their inhibition caused by metabolic disorders as lactic acidemia [14]. The high level of Interferon-γ in the first days after the clinical onset can also inhibit the differentiation of myeloid progenitors to eosinophils, thus favoring monocyte formation [22,23]. The cytokine storm mediated by T helper 1 lymphocytes could promote the rapid M1 polarization, lung migration, and accumulation of these cells [24,25].

Hematopoietic stem cells can also directly interact with the virus through direct infection, change in bone marrow environment, and/or through their Toll-Like Receptors. These interactions can cause impairment in cell differentiation mechanisms favoring, in turn, one or more specific lines [26]. The latter has been proposed as the mechanism underlying thrombocytopenia during SARS-1 [27]. Morphological abnormalities of platelets were described in COVID-19 in patients with both thrombocytosis and thrombocytopenia [28]. The authors explain this phenomenon with the severe, even if transient, perturbation of myelopoiesis caused in these patients by the cytokine storm. Morphological alterations were also identified in neutrophils, mainly including the production of immature cells, reduction in cytoplasmic granules, and Myeloperoxidase (MPO) [28]. These cells are of particular interest, because their count increased in both BAL and peripheral blood samples, together with monocytes, mainly in severe disease forms [24]. The pathogenic role of neutrophils in COVID-19 is likely due to the production of Neutrophil Extracellular Traps (NET), obtained with the release of DNA and MPO [28,29]. The titer of MPO-DNA was associated with a worse prognosis and increased inflammatory markers [29].

### 4.2. Study Limitations

Our study has some limitations. First of all, the retrospective nature could lead to unintentional missing data. In the present study, the score was retrospectively validated, whereas its effectiveness in real life should be prospectively proven. This is mainly true in patients with malignancy or autoimmune diseases, in which concomitant immunosuppressive treatment can favor the infection and, at the same time, alter the blood cell count [30]. The number of patients enrolled was relatively limited, and the control group was composed of infectious pneumonia in which a limited group of patients had atypical pneumonia. Moreover, only a small proportion of patients with CAP had been diagnosed with a viral etiology. For this reason, we cannot exclude that our score can be useful to detect viral pneumonia in general rather than only COVID-19. However, in these months of SARS-CoV-2 outbreak, most of the viral pneumonia are sustained by this virus, which represents a great concern for its management. On the other hand, it should be noted that 30% of our CAP patients showed imaging of atypical pneumonia, and most of our CAP patients had no identified pathogen. Therefore, it could be possible that at least some of our CAP patients could be sustained by a virus, alone or with a bacterial super-infection. [31]. The score was also derived in symptomatic patients with positive swabs: the performance in the diagnostic assessment of both oligo-asymptomatic COVID-19 subjects and swab-negative patients could be significantly different. Finally, our model is extremely complex and cannot be used in clinical practice without an available tool. To overcome this problem, we named our model the MECOR Score and developed an application available for common devices.

### 4.3. The MECOR Score in Real-Life and Study Strenghts

Before further discussion, it is necessary to highlight that the MECOR Score does not pretend to replace SARS-CoV-2 diagnosis by nasopharyngeal PCR testing but to support its diagnostic accuracy. In fact, one of the most significant challenges faced during the COVID-19 pandemic is to avoid misdiagnosis.

During the first stages of the COVID-19 outbreak, PCR test results often experienced severe delays if the number of tested patients exceeded laboratory capacity, leading to inappropriate patient placement. A widespread PCR testing shortage has also been confirmed by a recent trial in the United States, confirming that only 58% of patients received testing because of severe shortages [32]. This highlights the reason for which developing countries may suffer from shortages of PCR reagents and specialized laboratories, thus further delaying valuable test results. The WBC was recently included in an algorithm for the diagnosis of COVID-19, including High-Resolution Computed Tomography features [33]. Our model, being based solely on complete blood cell count, may be readily employed in countries with limited resources or cases when a prompt decision (pending test) should be taken (e.g., emergency medical and/or surgical conditions).

Moreover, despite the fact that the PCR test is considered the current gold standard for the diagnosis of COVID-19, this technique proved to have up to 30% false-negative results [34]. Therefore, positive patients can be admitted to a COVID-free ward contributing to sustained intrahospital transmission. If supported by external, prospective validation in this subgroup of patients, the MECOR score could be useful in the triage of patients with clinical presentation suggestive for COVID-19 supporting the PCR results. Our score could confirm a negative swab or, in case of high results, obtain a deeper respiratory sample (e.g., tracheal aspirate or BAL) or repeat the nasopharyngeal swab, before ruling out the patient as COVID-19 positive.

Regarding the MECOR Score performance, despite having calculated the most performing cut-off values (as shown in Table 5), we have plotted the probability of being COVID-19 positive according to each value of the MECOR Score (as shown in Figure 2). A given probability instead of a single number to rule-in or rule-out a specific event is indeed more helpful and could support the clinician in the decision to perform an invasive test if the probability is high. Cut-off values are always subject to false-positive and false-negative cases, and, sometimes, the clinical presentation may require more invasive tests, making cut-offs pointless [35].

## 5. Conclusions

This pilot retrospective study highlights the possibility to support a diagnosis of COVID-19 in the case of new waves of outbreak in patients with suggestive clinical presentation only evaluating Complete BloodCount. If validated in larger, prospective cohorts it could be useful in the case of new outbreak waves, supporting PCR tests in the case of delay or false negativity.

## Figures and Tables

**Table 1:** Demographics and laboratory characteristics among patients in the training and validation cohort. As auspicated, there was no statistically significant difference between the two cohorts.

	Training Cohortn *n* = 525	Validation Cohortn *n* = 250	Significance
Gender, Male (*n*, %)	331 (63%)	164 (65.6%)	NS
Age (years), Median (IQR)	64 (52–77)	63 (53–77)	NS
SARS-CoV-2 Positive Individuals, (*n*, %)	283 (53.9%)	135 (54%)	NS
CAP Pathogen			NE
Nonidentified	138 (57.1%)	69 (60%)	
*Streptococcus. pneumoniae*	35 (14.5%)	15 (13%)	
*Klebsiella pneumoniae*	20 (8.3%)	8 (7%)	
*Mycoplasma pneumoniae*	14 (5.8%)	6 (5.2%)	
Influenza	12 (5%)	7 (6%)	
*Mycobaterium tuberculosis*	6 (2.5%)	3 (2.6%)	
*Legionella. pneumophila*	5 (2%)	2 (1.7%)	
Metapneumovirus	3 (1.2%)	1 (0.9%)	
*Chlamydia* spp.	2 (0.82%)	1 (0.9%)	
*Haemophiluls influenzae*	2 (0.82%)	1 (0.9%)	
*Staphilococcus aureus*	2 (0.82%)	1 (0.9%)	
*Pseudomonas aeruginosa*	2 (0.82%)	1 (0.9%)	
Adenovirus	1 (0.41%)	0 (0%)	
WBC (cells/mm^3^), Median (IQR)	7900 (5400–12,080)	7800 (5300–11,800)	NS
NEUT (cells/mm^3^), Median (IQR)	6000 (3720–9520)	5910 (3600–9630)	NS
LYM (cells/mm^3^), Median (IQR)	970 (640–1470)	1010 (630–1495)	NS
MON (cells/mm^3^), Median (IQR)	510 (340–840)	530 (350–830)	NS
EOS (cells/mm^3^), Median (IQR)	0 (0–50)	0 (0–60)	NS
PLT (cells/µL), Median (IQR)	221,000 (159,000–295,000)	210,000 (156,000–294,000)	NS

CAP: Community-Acquired Pneumonia; IQR: interquartile range; WBC: white blood cell; NEUT: neutrophils; NS: not significant; NE: not evaluated; LYM: lymphocytes; MON: monocytes; EOS: eosinophils; PLT: platelets.

**Table 2:** Intracohort differences in demographics and laboratory characteristics.

**Training Cohort**			
	**SARS-CoV-2** **Negative** ***n* = 242**	**SARS-CoV-2** **Positive** ***n* = 283**	**Significance**
Gender, Male (*n*, %)	154 (63.6%)	177 (62.5%)	NS
Age (years), Median (IQR)	66 (50–79)	68 (57–77)	NS
WBC (cells/mm^3^), Median (IQR)	11,465 (8205–15,475)	5930 (4305–80,100)	*p* < 0.001
Patients with WBC < 4000 (cells/mm^3^), (*n*, %)	7 (2.9%)	60 (21.2%)	*p* < 0.001
NEUT (cells/mm^3^), Median (IQR)	8925 (5985–12,675)	4300 (2980–6415)	*p* < 0.001
Patients with NEUT < 1500 (cells/mm^3^), (*n*, %)	5 (2.1%)	9 (3.2%)	NS
LYM (cells/mm^3^), Median (IQR)	1185 (820–1768)	830 (505–1200)	*p* < 0.001
Patients with LYM < 1000 (cells/mm^3^), (*n*, %)	92 (38%)	178 (62.9%)	*p* < 0.001
MON (cells/mm^3^), Median (IQR)	755 (450–1048)	430 (300–600)	*p* < 0.001
Patients with MON < 200 (cells/mm^3^), (*n*, %)	11 (4.5%)	18 (6.4%)	NS
EOS (cells/mm^3^), Median (IQR)	20 (0–98)	0 (0–10)	*p* < 0.001
Patients with EOS < 50 (cells/mm^3^), (*n*, %)	151 (62.4%)	237 (83.7%)	*p* < 0.001
PLT (cells/µL), Median (IQR)	251,000 (191,000–330,000)	186,000 (144,500–258,000)	*p* < 0.001
Patients with PLT < 150,000 (cells/µL), (*n*, %)	33 (13.6%)	77 (27.2%)	*p* = 0.001
**Validation Cohort**			
	**SARS-Co-2V** **Negative** ***n* = 115**	**SARS-CoV-2** **Positive** ***n* = 135**	**Significance**
Gender, Male (n, %)	74 (64.3%)	90 (66.6%)	NS
Age (years), Median (IQR)	67 (55–77)	70 (54–78)	NS
WBC (cells/mm^3^), Median (IQR)	11,480 (8545–15,595)	5800 (4345–7600)	*p* < 0.001
Patients with WBC < 4000 (cells/mm^3^), (*n*, %)	2 (1.7%)	28 (20.7%)	*p* < 0.001
NEUT (cells/mm^3^), Median (IQR)	9190 (6325–12,865)	4180 (2995–5900)	*p* < 0.001
Patients with NEUT < 1500 (cells/mm^3^), (*n*, %)	3 (2.6%)	5 (3.7%)	NS
LYM (cells/mm^3^), Median (IQR)	1190 (780–1810)	880 (505–1200)	*p* < 0.001
Patients with LYM < 1000 (cells/mm^3^), (*n*, %)	44 (38.3%)	78 (57.7%)	*p* = 0.002
MON (cells/mm^3^), Median (IQR)	770 (420–110)	430 (305–600)	*p* < 0.001
Patients with MON < 200 (cells/mm^3^), (*n*, %)	9 (7.8%)	9 (6.6%)	NS
EOS (cells/mm^3^), Median (IQR)	30 (0–100)	0 (0–20)	*p* < 0.001
Patients with EOS < 50 (cells/mm^3^), (*n*, %)	67 (58.2%)	108 (80%)	*p* = 0.001
PLT (cells/µL), Median (IQR)	238,000 (184,500–317,000)	181,000 (144,000–251,000)	*p* < 0.001
Patients with PLT < 150,000 (cells/µL), (*n*, %)	18 (15.6)	37 (27.4%)	*p* = 0.025

IQR: interquartile range; NS: not significant; WBC: white blood cell; NEUT: neutrophils; LYM: lymphocytes; MON: monocytes; EOS: eosinophils; PLT: platelets.

**Table 3:** Univariate Analysis. Intracohort statistically significant values were studied univariately.

Univariate Analysis Training Set	(M1) WBC	(M2) NEUT	(M3) LYM	(M4) MON	(M5) EOS	(M6) PLT
Intercept (β_0_)	3.04	2.28	1.25	1.57	0.48	1.59
Intercept (β_0_)—Standard Error	0.28	0.23	0.19	0.19	0.1	0.24
Intercept (β_0_)—Significance	*p* < 0.001	*p* < 0.001	*p* < 0.001	*p* < 0.001	*p* < 0.001	*p* < 0.001
Coefficient (β_1_)	−3.24 × 10^−4^	−3.04 × 10^−4^	−9.65 × 10^−4^	−2.18 × 10^−3^	−8 × 10^−3^	−6 × 10^−6^
Coefficient (β_1_)—Standard Error	3.1 × 10^−5^	3.1 × 10^−5^	1.53 × 10^−4^	2.9 × 10^−4^	2 × 10^−3^	9.48 × 10^−7^
Coefficient (β_1_)—Significance	*p* < 0.001	*p* < 0.001	*p* < 0.001	*p* < 0.001	*p* < 0.001	*p* < 0.001
AUROC	0.82 (0.78–0.87)	0.80 (0.76–0.83)	0.68 (0.64–0.73)	0.72 (0.67–0.76)	0.71 (0.66–0.75)	0.67 (0.66–0.75)
Nagelkerke Pseudo-R2	0.410	0.350	0.140	0.190	0.011	0.120
Hosmer–Lemeshow *p*-Value	0.032	0.178	0.782	0.090	<0.001	0.450
AIC	538	572	668	649	684	679
BIC	546	580	677	657	692	689

WBC: white blood cell; NEUT: neutrophils; LYM: lymphocytes; MON: monocytes; EOS: eosinophils; PLT: platelets.

**Table 4:** Multivariate Model Information. The multivariate model was built employing those variables that univariately showed an AUROC > 0.65 and a Hosmer–Lemeshow *p*-Value > 0.05. Variables were transformed employing natural logarithmic and arctangent functions. Globally, the model was shown to be more balanced in its discrimination and calibration metrics if compared to each of the univariate models.

Multivariate Model	Variable 1 $\log_{e}\left(\mathrm{WBC}\right)$	Variable 2 $\log_{e}\left(\mathrm{LYM}\right)$	Variable 3 $\arctan\left(\frac{\mathrm{MON}\times \;\mathrm{NEUT}}{\mathrm{PLT}}\right)$
VIF	1.72	1.05	1.80
Coefficient	−3.23	−0.76	11.94
Coefficient—Standard Error	0.34	0.18	3.88
Coefficient—Significance	*p* < 0.001	*p* = 0.001	*p* = 0.002
Intercept	18.47		
Intercept—Standard Error	3.92		
Intercept—Significance	*p* < 0.001		
AUROC	0.86 (0.83; 0.90)		
Nagelkerke Pseudo-R2	0.5		
Hosmer-Lemeshow *p*-Value	0.9		
AIC	516		
BIC	533		

WBC: white blood cell; NEUT: neutrophils; LYM: lymphocytes; MON: monocytes; EOS: eosinophils; PLT: platelets.

**Table 5:** Cut-off Selection. Cut-off values were chosen in the training cohort in order to maximize either sensitivity or specificity or according to the Youden’s Index criteria. The same cut-off values were applied to the validation cohorts and showed comparable performance.

Cut-Off Parameter	Maximize Sensitivity ≤ −2.54		Maximize Specificity ≥ +4		Youden’s Index ≥ −0.13	
	Training	Validation	Training	Validation	Training	Validation
Sensitivity	100 (99–100)%	100 (92–100)%	2 (0.5–5)%	2 (0.5–7)%	84 (78–88)%	88 (81–93)%
Specificity	10 (6–15)%	12 (7–20)%	100 (99–100)%	100 (99–100)%	72 (67–77)%	73 (64–81)%
PPV	56 (55–57)%	58 (55–59)%	100%	100%	78 (74–81)%	79 (74–84)%
NPV	100%	100 %	47 (46–48)%	47 (45–48)%	79 (73–83)%	84 (76–90)%
+LR	1.11 (1.06–1.16)	1.14 (1.06–1.22)	0	0	2.92 (2.38–3.59)	3.27 (2.41–4.45)
−LR	0	0	0.98 (0.97–1)	0.98 (0.95–1)	0.23 (0.18–0.31)	0.16 (0.10–0.26)
Accuracy	59 (54–63)%	60 (60–66)%	47 (42–52)%	47 (40–54)%	80 (76–84)%	81 (76–86)%

PPV: positive predictive value; NPV: negative predictive value; +LR: positive likelihood ratio; −LR: negative likelihood ratio.

## Abbreviations

AIC	Akaike Information criterion
AUROC	Area Under Receiver-Operating Curve
BAL	Broncho-Alveolar Lavage
BIC	Bayesian Information Criterion
CAP	Community-Acquired Pneumonia (CAP)
COVID-19	COronaVirus Disease 2019
EOS	Eosinophils
ER	Emergency Rooms (ER)
HRCT	High-Resolution Computed Tomography
LP	Linear predictor
LR	Likehood Ratio
LYM	Lymphocytes
MECOR	Model for Early COvid-19 Recognition
MON	Monocytes
MPO	Myeloperoxidase
NET	Neutrophil Extracellular Trap
NEUT	Neutrophils
NLR	Neutrophil-to-Lymphocyte Ratio (NLR)
NPV	Negative Predictive Value
PLT	platelets
PPV	Positive Predictive Value
RT-PCR	real-time reverse-transcription polymerase chain reaction
SARS-CoV-2	Severe Acute Respiratory Syndrome-CoronaVirus 2
VIC	Cariance inflaction factor
WBC	White Blood Cells
WHO	World Health Organization
